# Supplementary material for: Discovery of senolytics using machine learning
Source: Nat Commun. 2023 Jun 10;14:3445. doi: 10.1038/s41467-023-39120-1 (PMC10257182; doi:10.1038/s41467-023-39120-1)
Supplement: Supplementary file 1 — Supplementary Information [file 41467_2023_39120_MOESM1_ESM.pdf]

Supplementary Material

Discovery of senolytics using machine learning

Vanessa Smer-Barreto[1,#,*], Andrea Quintanilla[2,#], Richard J. R. Elliott[1], John C. Dawson[1], Jiugeng Sun[3], Víctor M. Campa[2], Álvaro Lorente-Macías[1], Asier Unciti-Broceta[1], Neil O. Carragher[1], Juan Carlos Acosta[1,2,*], Diego A. Oyarzún[3,4,5,*].

[1] Cancer Research UK Edinburgh Centre, MRC Institute of Genetics and Cancer, University of Edinburgh, Crewe Road, Edinburgh, EH4 2XR, UK.
[2] Instituto de Biomedicina y Biotecnología de Cantabria (IBBTEC), CSIC-Universidad de Cantabria-SODERCAN. C/ Albert Einstein 22, Santander, 39011, Spain.
[3] School of Informatics, University of Edinburgh, 10 Crichton St, Edinburgh, EH8 9AB, UK.
[4] School of Biological Sciences, University of Edinburgh, Max Born Crescent, Edinburgh, EH9 3BF, UK.
[5] The Alan Turing Institute, 96 Euston Road, London, NW1 2DB, UK.

[*] Corresponding authors: vanessa.smerbarreto@ed.ac.uk; juan.acosta@unican.es; d.oyarzun@ed.ac.uk

Supplementary Figures

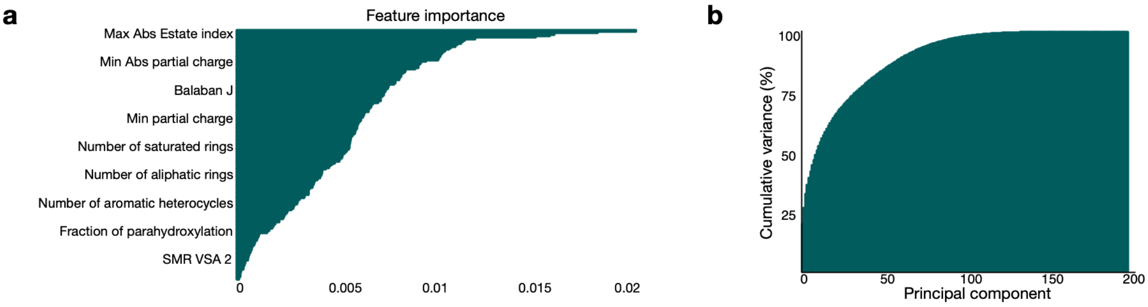

Supplementary Figure 1. Evaluation of feature importance for training of machine learning models. (a) Features used for training of machine learning models from RDKit, ranked by feature importance percentage, using the Mean Decrease Gini as the impurity measure (definition in Methods and Materials Section B).165 descriptors out of 200 calculated gave a non-zero importance score, and were therefore utilised to train the machine learning models described in Fig. 2 and Supplementary Table 2. **(b)** Cumulative variance per feature of the original set of 200 RDKit features computed out of the data set. To explain 99% of the variance in the data set, 111 features are needed. This points at a problem of incompressibility of this chemical space. A description of these descriptors may be found on the RDKit website [57]. Source data are provided as a Source Data file.

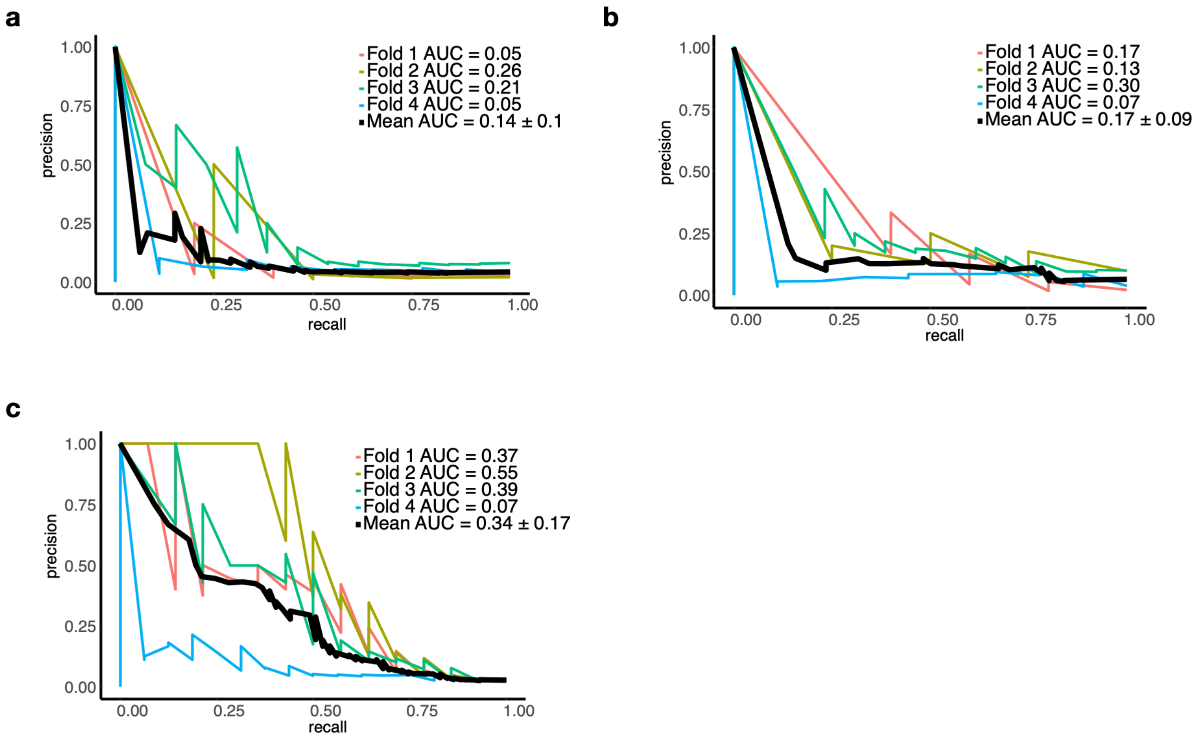

Supplementary Figure 2. Comparison of XGBoost model against a deep message-passing neural network (D-MPNN). (a) Individual and mean precision-recall curves in 4-fold cross-validation for D-MPNN trained on atom/bond standard model features. **(b)** Individual and mean precision-recall curves for D-MPNN in 4-fold cross-validation trained on atom/bond standard model features plus molecular level RDKit 2D features. **(c)** Individual and mean precision-recall curves of XGBoost model in main text with 4-fold cross-validation trained on RDKit 2D features. D-MPNN models were trained with the chemprop library (https://github.com/chemprop/chemprop). In all three cases, 80% of the data was used to train each fold. Source data are provided as a Source Data file.

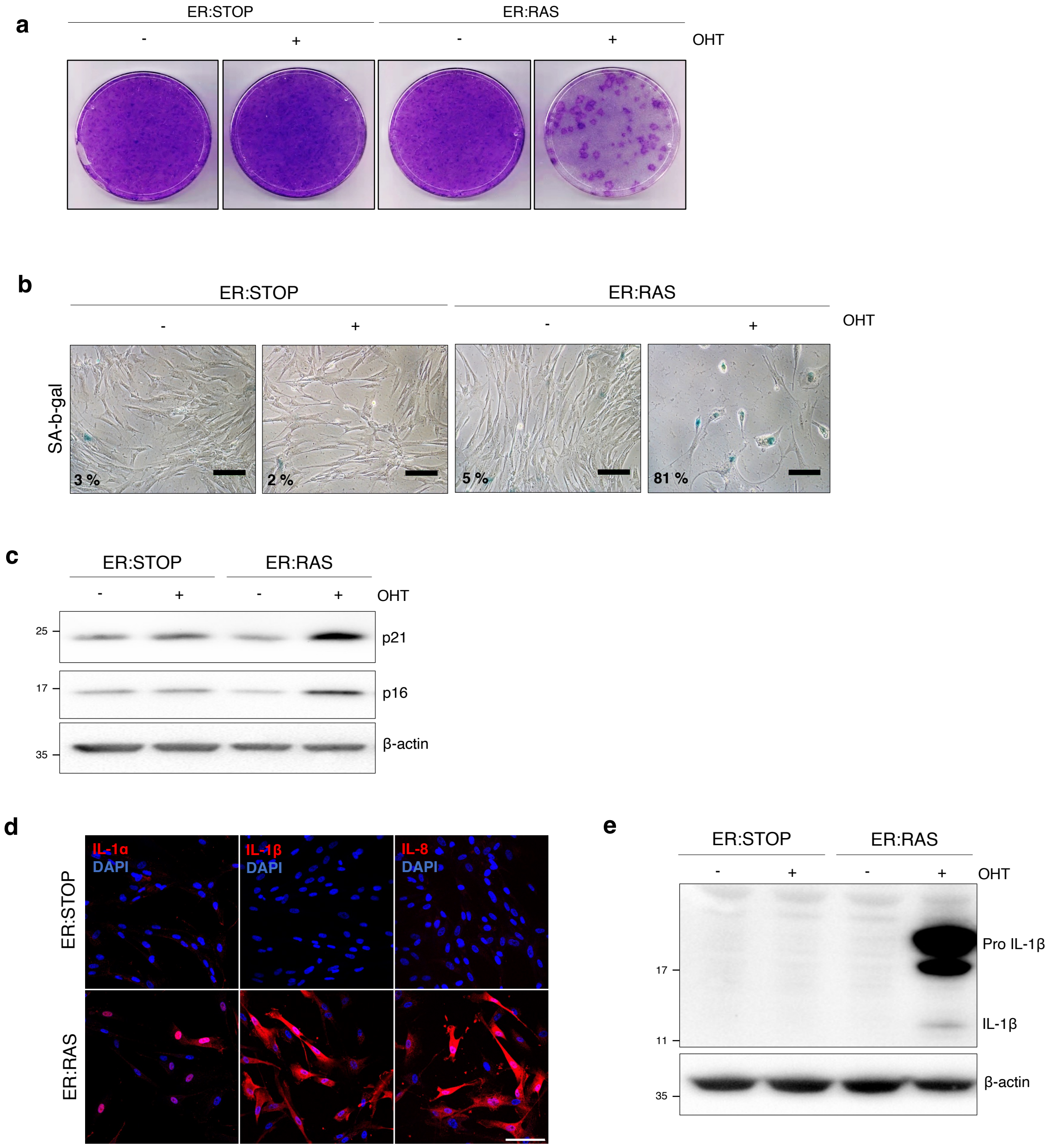

Supplementary Figure 3. Oncogene-induced senescence model (OIS) characterization in human diploid fibroblasts IMR90. (a) Cell proliferation assay of IMR90 ER:STOP and ER:RAS cells. Cells plated at the same density and cultured, with 100 nM 4OHT or not as indicated for 12 days and then stained for crystal violet. **(b)** SA-β-galactosidase activity assay of IMR90 ER:STOP and IMR90 ER:RAS cells cultured with 4OHT or not as indicated, performed as Methods. Percentage of SA-β-galactosidase positive cells per condition is indicated. Scale bars represent 100 µm. **(c)** Western blot of p21 and p16 expression in IMR90 ER:STOP and ER:RAS cells treated with 4OHT as indicated for 4 days. β-actin is shown as loading control. **(d)** Immunofluorescence labelling for IL-1α, IL-1β and IL-8 of IMR90 ER:STOP and ER:RAS cells cultured with 4OHT 100 nM for 8 days. Representative images obtained in a confocal microscope are shown. Scale bars represent 100 µm. **(e)** Western blot of IL-1β expression in IMR90 ER:STOP and ER:RAS cells cultured with 4OHT as indicated for 8 days. β-actin is shown as loading control. All panels are representative images of experiments that have been repeated independently five times with similar results.

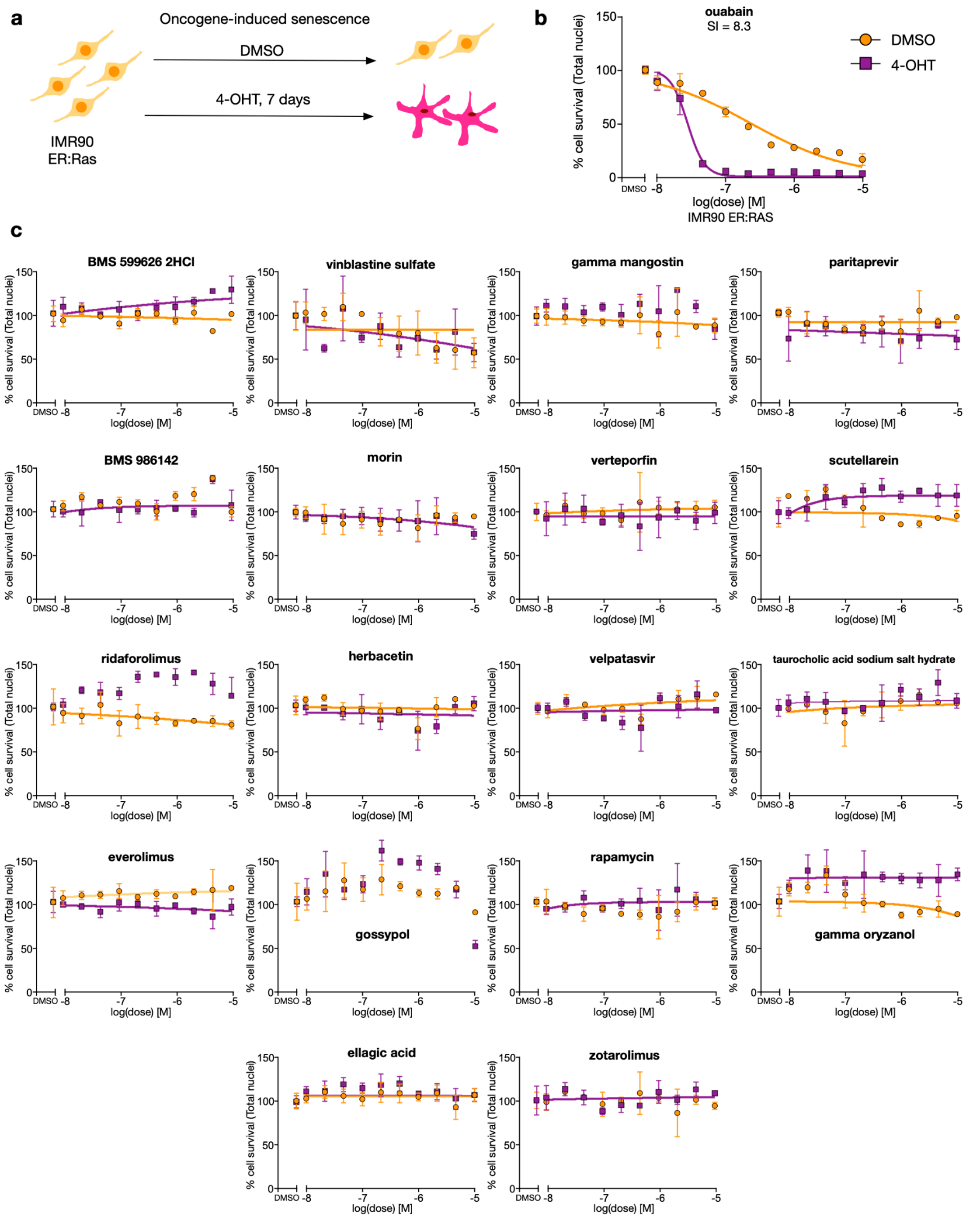

Supplementary Figure 4. Experimental characterisation of false positives predicted by the machine learning model in oncogene-induced senescence. (a) Experimental setup of oncogene-induced senescence model with IMR90 ER:RAS cells. Senescence was induced by addition of 4-OHT at 100 nM for six days. Control and senescent cells were plated on the last day of senescence induction. Top predicted compounds were added a day later, and 72 hours afterwards, the cells were fixed, and the nuclei stained and counted. **(b)** Dose-response curve of OIS positive experimental control, ouabain. Data is normalised to DMSO. Mean ± s.d. are shown from n=3 experiments. **(c)** Dose-response curves of false positives of the XGBoost model (non-senolytic compounds predicted to have senolytic action with P>44%). Data is normalised to DMSO. Mean ± s.d. are shown from n=3 experiments. Source data are provided as a Source Data file.

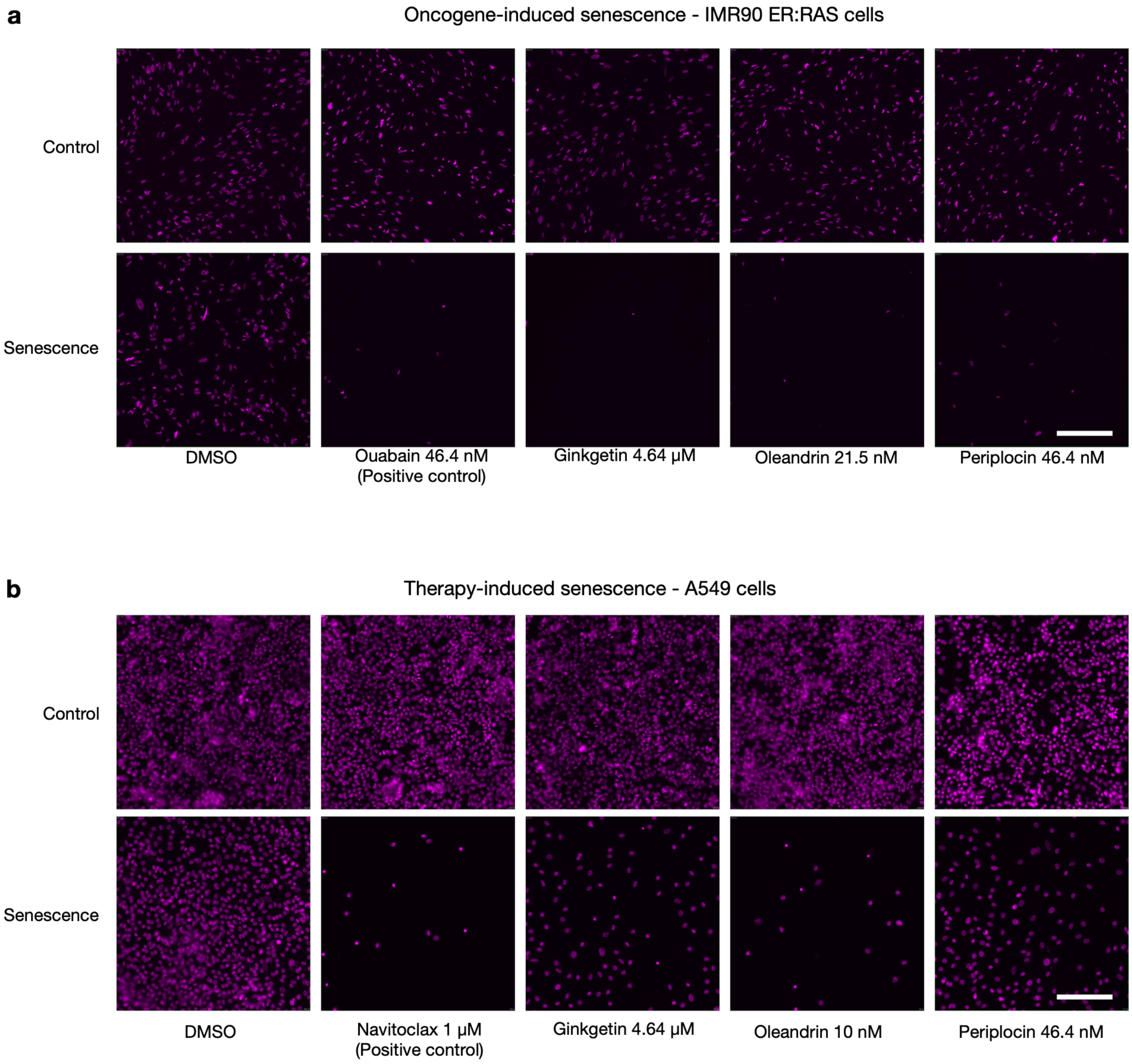

Supplementary Figure 5. Representative images of nuclei for controls and newly found senolytics from drug screen shown in figure 3C and Supplementary Figure 6C. (a) 10× images of IMR90 ER:RAS cell nuclei of oncogene-induced senescence experiment. The positive control and the three found senolytics images are displayed at the minimal concentration where the senolytic effect was first observed (violet stain is DAPI channel). Scale bar is 100 μM. Representative images from the screen in Figure 3C, which was done once with three experimental replicates. **(b)** 20× images of A549 cell nuclei of therapy-induced senescence experiment. The positive control and the three found senolytics images are displayed at the minimal concentration where the senolytic effect was first observed (violet stain is DAPI channel). Scale bar is 50 μM. Representative images from the screen in Supplementary Figure 6C, which was done once with three experimental replicates.

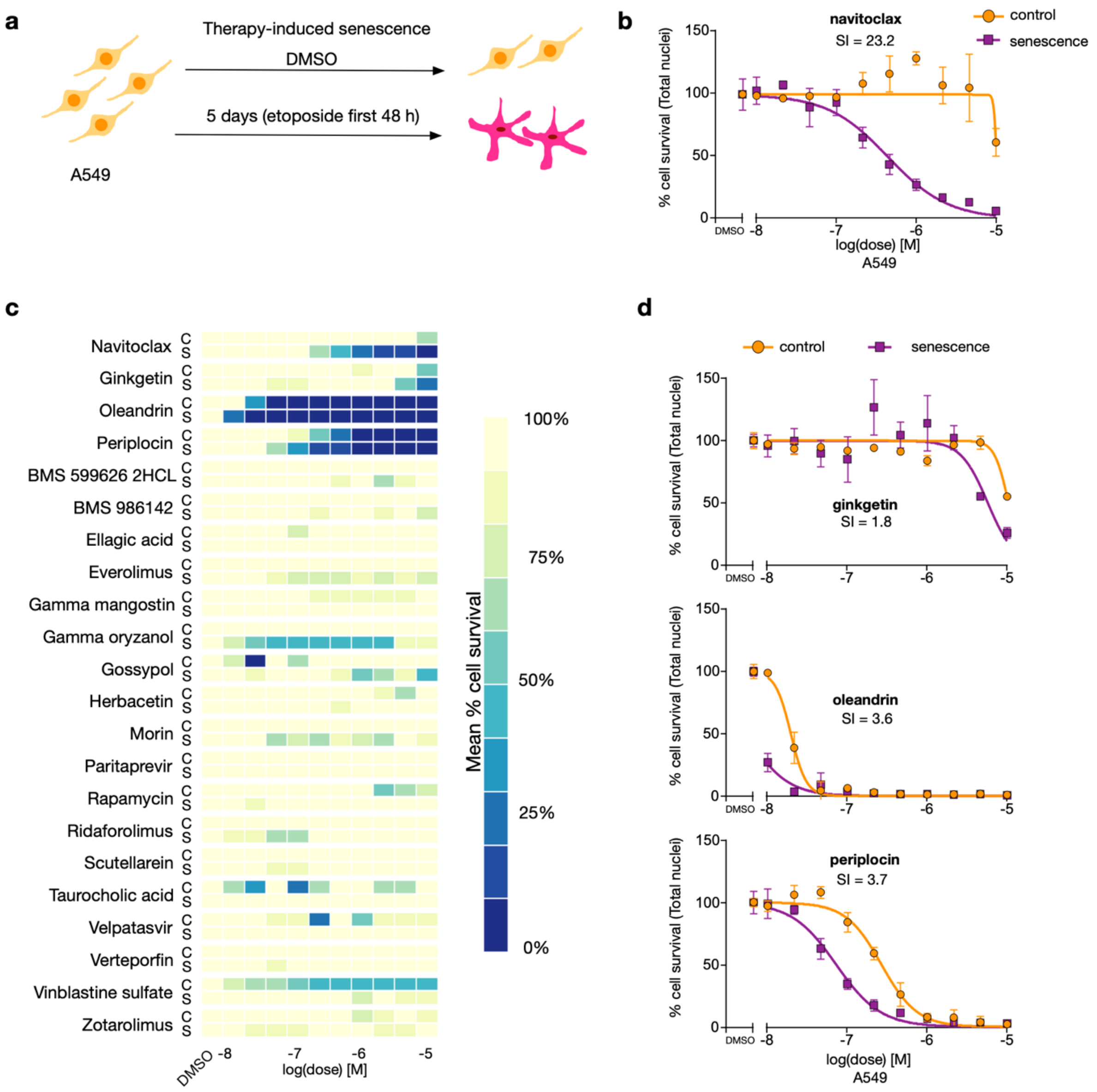

Supplementary Figure 6. Experimental characterisation of compounds selected for screening in therapy-induced senescent cells. (a) Experimental setup of therapy-induced senescence model with A549 cells. Senescence was induced by addition of etoposide at 100 μM for 48 hours, followed by three subsequent days of culturing in standard conditions. Control and senescent cells were plated on the last day of senescence induction. Top predicted compounds were added a day later, and 72 hours afterwards, the cells were fixed, and the nuclei stained and counted. **(b)** Dose-response curve of TIS positive experimental control, navitoclax. Data is normalised to DMSO. Mean ± s.d. are shown from n=3 experiments. **(c)** Results from experimental validation of controls and top 21 compounds predicted to have senolytic action with P>44%; heatmap shows mean across n=3 experimental replicates. **(d)** Dose-response curves of the three compounds out of 21 that displayed senolytic activity: ginkgetin, oleandrin, and periplocin. Data is normalised to DMSO. Mean ± s.d. are shown from n=3 experiments. Source data are provided as a Source Data file.

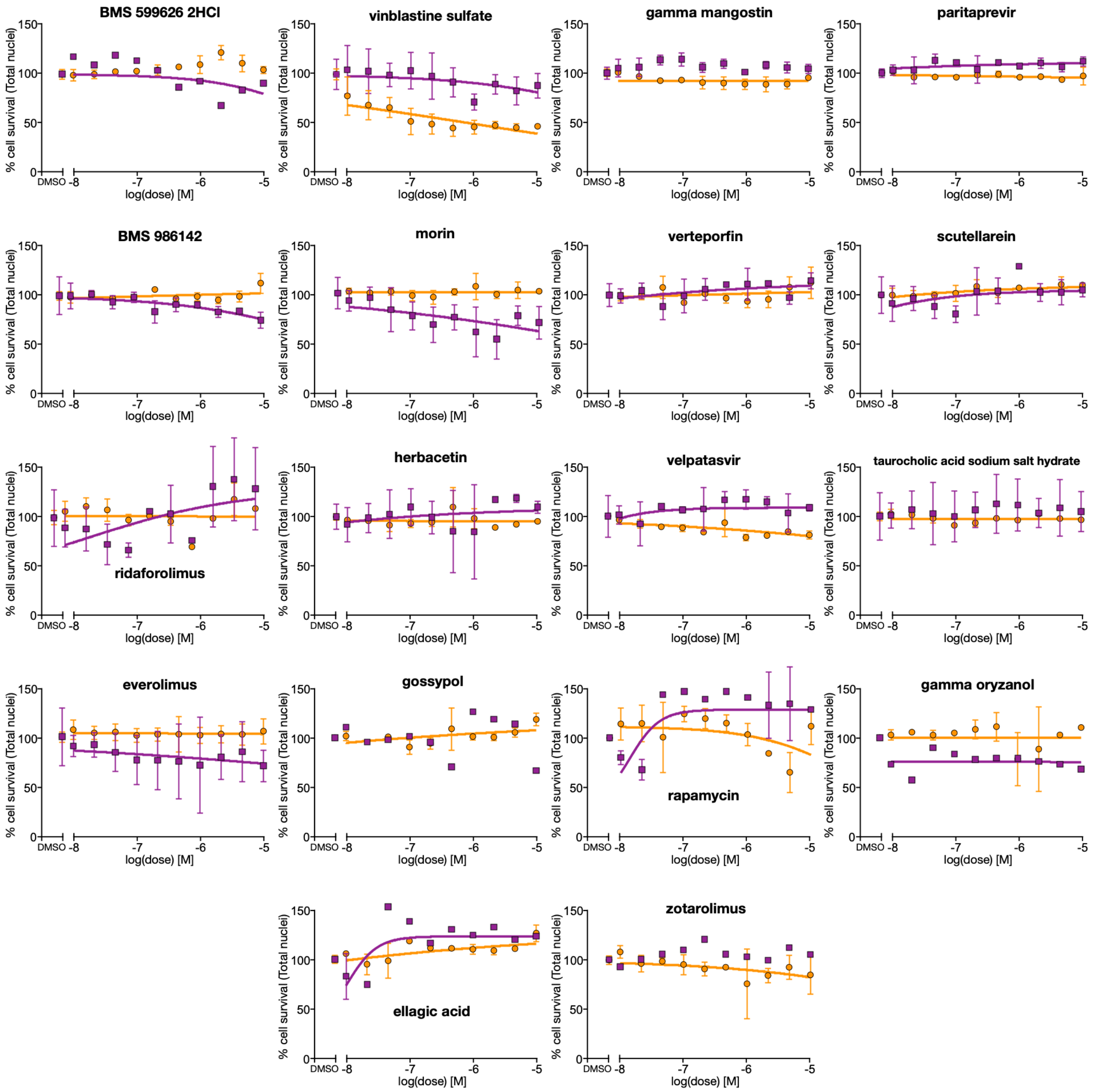

Supplementary Figure 7. Experimental characterisation of false positives predicted by the machine learning model in therapy-induced senescent cells. Dose-response curves of non-senolytic compounds predicted to have senolytic action by the XGBoost model with P>44%. Data is normalised to DMSO. Mean ± s.d. are shown from n=3 experiments. Source data are provided as a Source Data file.

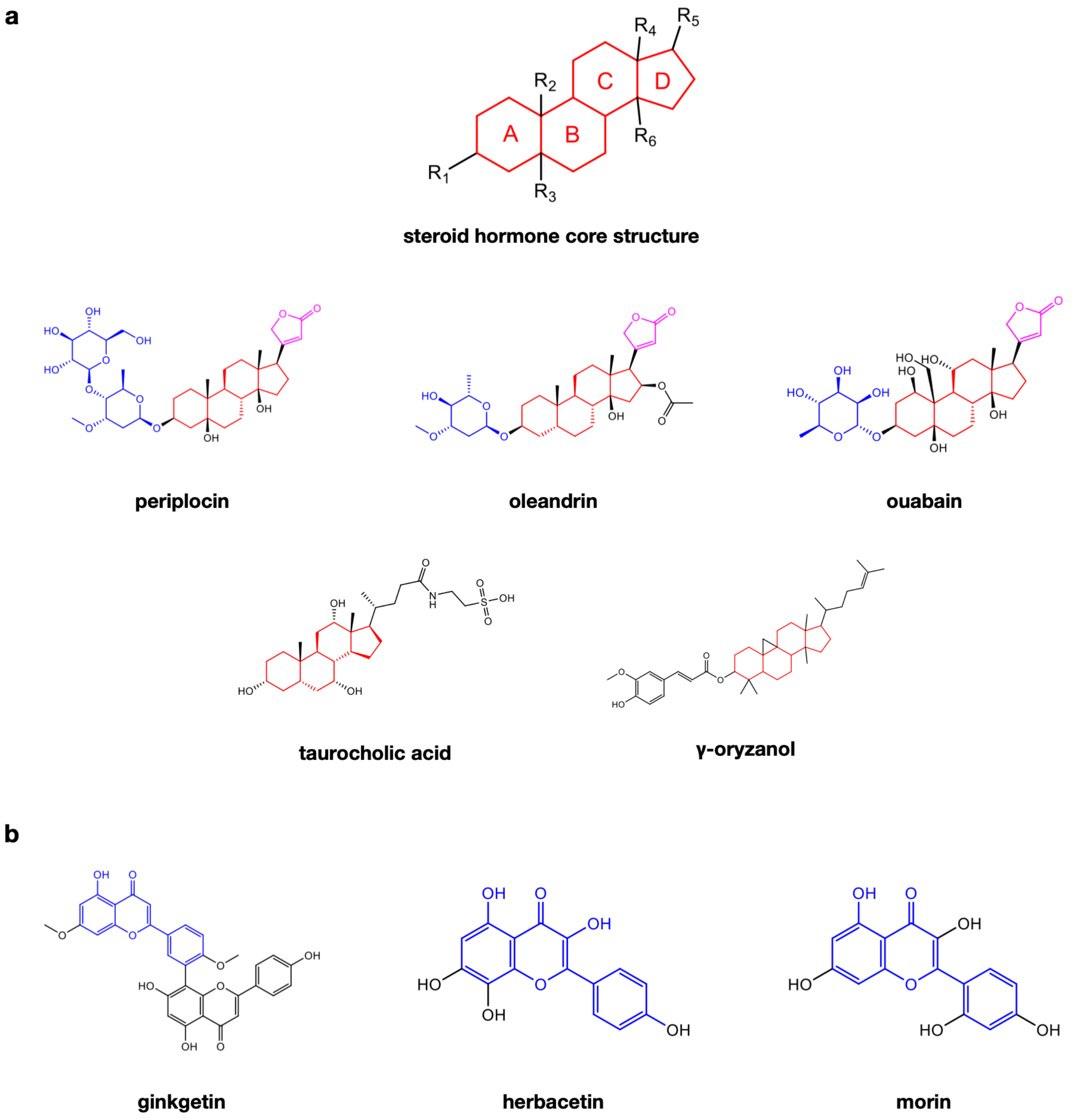

Supplementary Figure 8. Chemical description of molecules present in experimental screen. (a) The steroid hormone core structure alone (coloured red) present in periplocin, oleandrin and ouabain is insufficient for senolytic activity, when compared to inactive compounds, taurocholic acid and gamma-oryzanol. Hence the glycoside linkages (coloured blue) and furanone moiety (purple) are clearly contributing to target binding and potency, beyond physicochemical properties (solubility, cell permeability, etc). **(b)** The basic flavone scaffold present in ginkgetin (coloured blue) is also present in inactives, herbacetin and morin. This may indicate a more complex target binding mode for the asymmetrical biflavone unit of ginkgetin, and is worth further investigation.

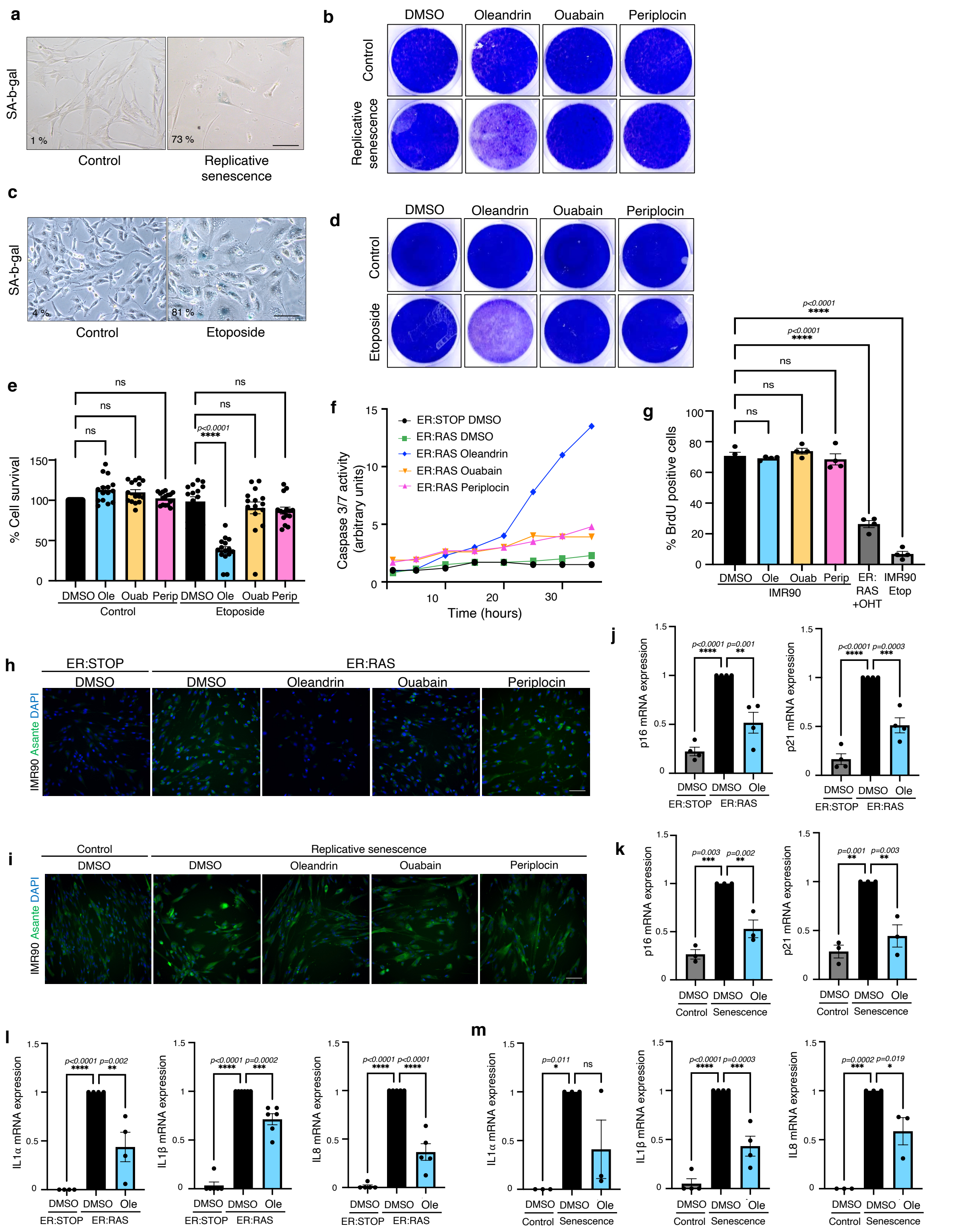

Supplementary Figure 9. Oleandrin and periplocin senolytic performance on several senescence models. (a) SA-β-galactosidase activity assay, performed as Methods, of proliferating IMR90 cells (passage 13) (control) and senescent IMR90 at passage 27 (replicative senescence). Percentage of SA-β-galactosidase positive cells per condition is indicated. **(b)** Cell survival assay measuring the senolytic effect in IMR90 replicative senescence model. The panels show a representative crystal violet staining of tissue culture dishes of confluent senescent IMR90 at passage 27 and control IMR90 at passage 13 cells treated with 10 nM oleandrin, ouabain and periplocin, and DMSO as vehicle control for 72 hours. Graph representing the cell survival by quantification of the crystal violet staining of the experiment is shown in Fig. 4c. **(c)** SA-β-galactosidase activity assay of A549 cells treated with vehicle (control) and A549 treated with 100 μM etoposide (etoposide). Percentage of SA-β-galactosidase positive

cells per condition is indicated. **(d)** Cell survival assay measuring the senolytic effect in lung cancer senescent cells. The panels show a representative crystal violet staining of tissue culture dishes of confluent A549 cells treated with 100 µM etoposide and control A549 cells, all treated with 10 nM oleandrin, ouabain and periplocin, and DMSO as vehicle control for 72 hours as indicated. **(e)** Graph representing the cell survival by quantification of the crystal violet staining of the experiment described in (d), as described in Methods. Data represented as individual datapoints, and bars and error bars representing the mean ± SEM of 14 independent experiments. Statistical analysis was performed using a one-way ANOVA (Tukey's test) for multiple comparisons. **(f)** Time-lapse imaging of caspase3/7 activity assay in control IMR90 ER:STOP and senescent IMR90 ER:RAS cells cultured in media containing 100 nM 4OHT, and treated during 35 hours with 10 nM oleandrin, ouabain and periplocin, and DMSO as vehicle control. Representative fluorescent images of caspase 3/7 positive cells and brightfield images of the same field for cells coring are shown in Fig. 4d. Representative data of one of two independent experiments. **(g)** Quantification of BrdU immunofluorescence staining of IMR90 cells treated with 10 nM oleandrin, ouabain and periplocin, and DMSO as vehicle. BrdU quantification was performed in IMR90 cells treated with etoposide and 100 nM 4OHT treated IMR90 ER:RAS as experimental controls. Data represented as individual data points, and bars and error bars representing the mean ± SEM of 4 independent experiments. Statistical analysis was performed using a one-way ANOVA (Tukey's test) for multiple comparisons. **(h)** Representative images from intracellular K+ levels measured using Asante staining in 100 nM 4OHT containing cultures of senescent IMR90 ER:RAS cells treated with 10 nM oleandrin, ouabain and periplocin, or DMSO as vehicle control, compared with IMR90 ER:STOP controls, as shown in Fig. 5a. **(i)** Representative images from intracellular K+ levels measured using Asante staining in IMR90 cells at passage 27 (replicative senescence) treated with 10 nM oleandrin, ouabain and periplocin, or DMSO as control vehicle compared to IMR90 cells at passage 13 (control), as shown in Fig. 5b. **(j-k)** mRNA expression of p16 and p21 determined by RT-qPCR in **(j)** 100 nM 4OHT containing cultures of IMR90 ER:STOP and IMR90 ER:RAS cells treated with 10 nM oleandrin and DMSO as vehicle control (n=4), and **(k)** IMR90 cells at passage 27 (replicative senescence) treated with 10 nM oleandrin and DMSO control compared to IMR90 proliferating cells at passage 13 (control) (n=3). Data represented as individual data points and bars and error bars representing the mean ± SEM. Statistical analysis was performed using one-way ANOVA (Dunnett's test) for multiple comparisons. **(l-m)** mRNA expression of IL1α, IL1β and IL8 determined by RT-qPCR in **(l)** 100 nM 4OHT containing cultures of IMR90 ER:STOP and IMR90 ER:RAS cells treated with 10 nM oleandrin and DMSO as vehicle control (IL1α n=4, IL1β n=6, IL8 n=5), and **(m)** IMR90 cells at passage 27 (replicative senescence) treated with 10 nM oleandrin and DMSO control, compared to IMR90 proliferating cells at passage 13 and treated with DMSO (control) (IL1α n=3, IL1β n=4, IL8 n=3). Data represented as individual data points and bars and error bares representing the mean ± SEM. Statistical analysis was performed using one-way ANOVA (Dunnett's test) for multiple comparisons. Scale bars represent 100 µm. ns = not significant, * = $p < 0.05$, ** = $p < 0.01$, *** = $p < 0.001$, **** = $p < 0.0001$. Source data are provided as a Source Data file.

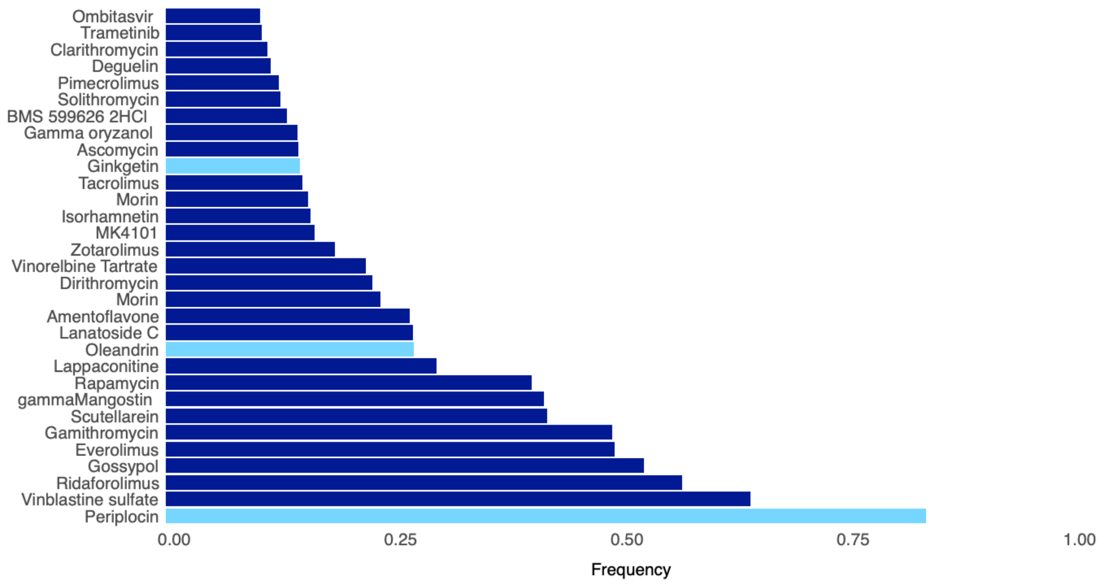

Supplementary Figure 10. Robustness of predicted hits by the computational screen. To investigate the robustness of our hits selected for experimental validation (Fig. 2d-e), we retrained the XGBoost model with resampled training data 1,000 times, and computed the number of instances a compound in the screening library was assigned a prediction score P>44%. Bar plots show the frequency of compounds with predicted P>44% across the 1,000 repeats between training and screening. The XGBoost hyperparameters were kept constant for all repeats (Supplementary Table 5). Our three validated hits are consistently assigned P>44% by the model. Source data are provided as a Source Data file.

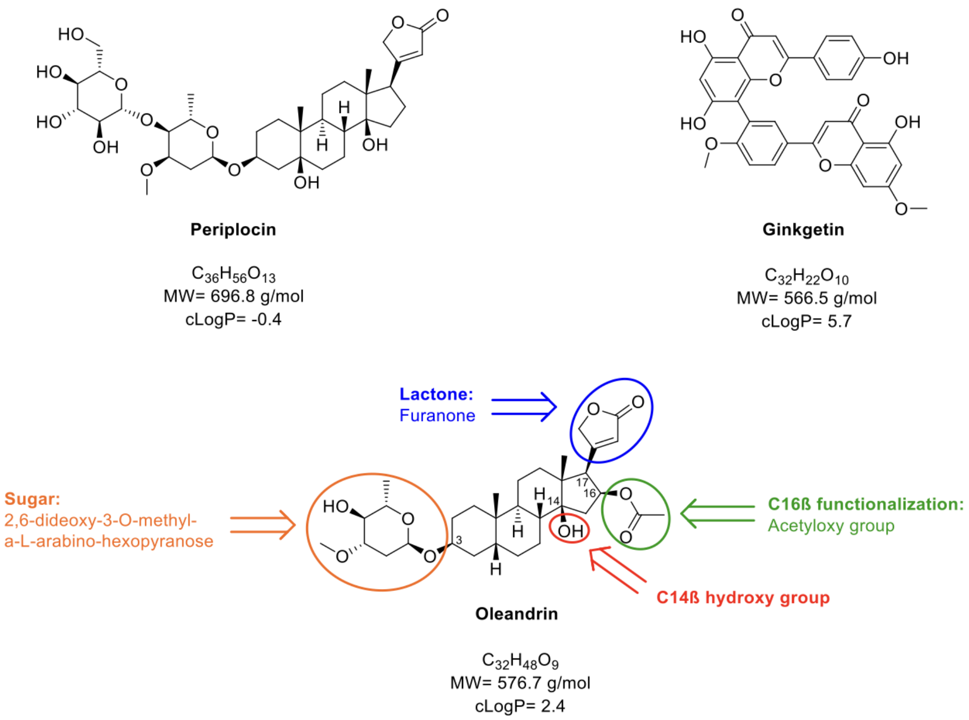

Supplementary Figure 11. Chemical structure of periplocin, ginkgetin and oleandrin. Structural motifs of oleandrin are highlighted as coloured circles.

Supplementary Figure 3c

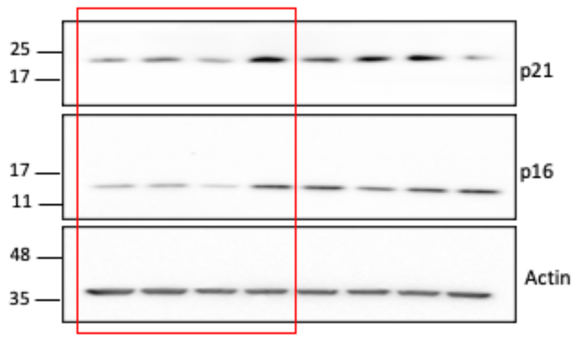

Supplementary Figure 3e

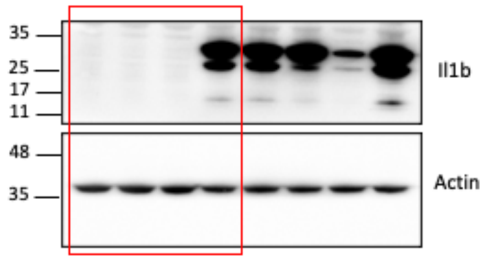

Supplementary Figure 12. Full scan immunoblots used in figures.

Supplementary Tables

Model	Precision	Recall	F_1 score
Random forest with class weight None	0.63 ± 0.37	0.08 ± 0.05	0.2 ± 0.06
SVM with class weight balanced and linear kernel	0.11 ± 0.03	0.58 ± 0.25	0.19 ± 0.06
XGBoost with max depth = 10	0.57 ± 0.39	0.16 ± 0.12	0.24 ± 0.17

Supplementary Table 1. Performance metrics of binary classifiers trained on 200 RDKit features. The most successful algorithms (SVM, RF, XGBoost) were trained with the full set of 200 features to test whether there was loss in prediction accuracy due to feature selection. These performance metrics were calculated utilising 5-fold cross-validation on the whole dataset of 2,523 rows and 200 selected features, we are reporting the mean and standard deviation of this procedure. The performance metrics for two of the models, random forest and XGBoost, are worse than when utilising a reduced set of features.

Model	Precision	Recall	F_1 score
K-nearest neighbours (K=5)	0.52 ± 0.32	0.12 ± 0.09	0.19 ± 0.13
Gaussian Bayes	0.04 ± 0.02	0.63 ± 0.14	0.09 ± 0.04
Logistic regression	0.2 ± 0.4	0.02 ± 0.03	0.03 ± 0.06
Decision tree	0.3 ± 0.08	0.39 ± 0.13	0.33 ± 0.1
Voting classifier (RF, SVM, XGBoost)	0.6 ± 0.49	0.09 ± 0.08	0.18 ± 0.11
Stacking ensemble (Estimators: RF, SVM, XGBoost; Final estimator: logistic regression)	0.77 ± 0.3	0.1 ± 0.06	0.18 ± 0.05
SMOTE + Tomek links with SVM	0.25 ± 0.18	0.2 ± 0.17	0.21 ± 0.18
Random forest with class weight None	0.73 ± 0.38	0.15 ± 0.06	0.18 ± 0.11
SVM with class weight balanced and linear kernel	0.1 ± 0.02	0.51 ± 0.14	0.17 ± 0.03
XGBoost with max depth = 10	0.7 ± 0.16	0.24 ± 0.09	0.35 ± 0.01

Supplementary Table 2. Performance metrics of other classifiers trained on the assembled dataset of senolytics. Besides the models in the main text (SVM, RF, XGBoost), we trained a variety of other models on 2,523 compounds using the 165 selected features; we report the mean and standard deviation of each metric across 5-fold cross-validation. We also trained various models with data pre-processed with SMOTE [84], a method to resample imbalanced datasets, but the results were generally poor (table reports the best SMOTE model only); SMOTE was employed on the training set only. Several models give values of precision = recall = 0 when applying cross-validation and performing grid searches for hyperparameter tuning due to the heavy class imbalance in the dataset. In general, the performance of these additional models was consistently poorer than the models reported in the main text (Fig 2b-c). Additionally, we trained a deep-message passing neural network (D-MPNN [39,47]) on atom and edge representations, which also showed poorer cross-validation performance than the XGBoost model (Supplementary Figure 2).

Hyperparameter	Value
Regularisation parameter	1.0
Kernel	Linear
Shrinking heuristic	True
Probability	True
Tolerance for stopping criterion	1e-3
Cache size	200
Class weight	balanced
Verbose	False
Maximal iterations	-1
Decision function shape	ovr
Break ties	False
Random state	None

Supplementary Table 3. Optimised hyperparameters for support vector machine (SVM) model produced by 5-fold cross-validation.

Hyperparameter	Value
Bootstrap	True
CCP Alpha	0
Class weight	None
Criterion	gini
Max depth	None
Max features	auto
Max leaf nodes	None
Max samples	None
Min impurity decrease	0
Min impurity split	None
Min samples leaf	1
Min samples split	2
Min weight fraction leaf	0
Number of estimators	100
Number of jobs	None
Out of bounds score	False
Random state	None
Verbose	0
Wam start	False

Supplementary Table 4. Optimised hyperparameters for random forest (RF) model produced by 5-fold cross-validation.

Hyperparameter	Value
Base score	0.5
Booster	gbtree
Col sample by level	1
Col sample y node	1
Col sample by tree	0.5
Gamma	0
Learning rate	0.5
Max delta step	0
Max depth	10<br>(determined by grid search in the interval [1,10])
Min child weight	1
Missing	None
Number of estimators	100
Number of jobs	1
Nthread	None
Objective	binary:logistic
Random state	0
Reg alpha	0
Reg lambda	1
Scale positive weight	1
Seed	None
Silent	None
Subsample	1
Verbosity	1

Supplementary Table 5. Choice of hyperparameters for XGBoost algorithm using 5-fold cross-validation.
