# Supplementary material for: Discovery of senolytics using machine learning
Source: Nat Commun. 2023 Jun 10;14:3445. doi: 10.1038/s41467-023-39120-1 (PMC10257182; doi:10.1038/s41467-023-39120-1)
Supplement: Supplementary file 3 — Reporting Summary [file 41467_2023_39120_MOESM3_ESM.pdf]

nature portfolio

Corresponding author(s): Vanessa Smer-Barreto, Juan Carlos Acosta, Diego Oyarzun

Last updated by author(s): May 23, 2023

Reporting Summary

Nature Portfolio wishes to improve the reproducibility of the work that we publish. This form provides structure for consistency and transparency in reporting. For further information on Nature Portfolio policies, see our Editorial Policies and the Editorial Policy Checklist.

Statistics

For all statistical analyses, confirm that the following items are present in the figure legend, table legend, main text, or Methods section.

n/a	Confirmed	
☐	☒	The exact sample size (n) for each experimental group/condition, given as a discrete number and unit of measurement
☐	☒	A statement on whether measurements were taken from distinct samples or whether the same sample was measured repeatedly
☐	☒	The statistical test(s) used AND whether they are one- or two-sided<br>*Only common tests should be described solely by name; describe more complex techniques in the Methods section.*
☐	☒	A description of all covariates tested
☒	☐	A description of any assumptions or corrections, such as tests of normality and adjustment for multiple comparisons
☐	☒	A full description of the statistical parameters including central tendency (e.g. means) or other basic estimates (e.g. regression coefficient) AND variation (e.g. standard deviation) or associated estimates of uncertainty (e.g. confidence intervals)
☐	☒	For null hypothesis testing, the test statistic (e.g. F, t, r) with confidence intervals, effect sizes, degrees of freedom and P value noted<br>*Give P values as exact values whenever suitable.*
☒	☐	For Bayesian analysis, information on the choice of priors and Markov chain Monte Carlo settings
☒	☐	For hierarchical and complex designs, identification of the appropriate level for tests and full reporting of outcomes
☒	☐	Estimates of effect sizes (e.g. Cohen's d, Pearson's r), indicating how they were calculated

Our web collection on statistics for biologists contains articles on many of the points above.

Software and code

Policy information about availability of computer code

Data collection

Time-lapse imaging was recorded a Leica AF6500 microscope (10x). DAPI staining was acquired in a Nikon TI2 microscope (20x). Screen imaging was performed in an ImageXpress Micro XLS (Molecular Devices). Confocal images of autofluorescence staining were acquired on a SP5 laser-scan microscope (Leica) with a 20× NA objective and 2× electronic zoom using LAS AF acquisition software.

Data analysis

GraphPad Prism 6 was used to fit the dose-response curves for all compounds. GraphPad Prism 9 was used for statistical analyses. The R package rstatix (0.7.0) was used for the statistical analysis in Fig 3b. SMILES strings were computed with ChemDraw 18.1.0.535 and chemical descriptors were computed with the RDKit package. Image analysis was performed on MetaXpress v6.6.2.46 and ImageJ 1.53k.

Python code for model training and computational screening is available at https://doi.org/10.5281/zenodo.7870357. The code has been commented extensively. To ensure reproducibility, we have also provided the curated training data and screening library as csv files.

Python package list: Python (3.8.3), seaborn (0.10.0), numpy (1.18.1), pandas (1.0.1), matplotlib (3.1.3), sklearn (0.24.1), pickle (4.0), xgboost (0.90)

For manuscripts utilizing custom algorithms or software that are central to the research but not yet described in published literature, software must be made available to editors and reviewers. We strongly encourage code deposition in a community repository (e.g. GitHub). See the Nature Portfolio guidelines for submitting code & software for further information.

Data

Policy information about availability of data

All manuscripts must include a data availability statement. This statement should provide the following information, where applicable:

- Accession codes, unique identifiers, or web links for publicly available datasets
- A description of any restrictions on data availability
- For clinical datasets or third party data, please ensure that the statement adheres to our policy

Data for model training and computational screen is available at https://doi.org/10.5281/zenodo.7870357. Data for in-vitro biological experiments is included in the 'source data' supplementary file.

Research involving human participants, their data, or biological material

Policy information about studies with human participants or human data. See also policy information about sex, gender (identity/presentation), and sexual orientation and race, ethnicity and racism.

Reporting on sex and gender	Not applicable
Reporting on race, ethnicity, or other socially relevant groupings	Not applicable
Population characteristics	Not applicable
Recruitment	Not applicable
Ethics oversight	Not applicable

Note that full information on the approval of the study protocol must also be provided in the manuscript.

Field-specific reporting

Please select the one below that is the best fit for your research. If you are not sure, read the appropriate sections before making your selection.

- [x] Life sciences
- [ ] Behavioural & social sciences
- [ ] Ecological, evolutionary & environmental sciences

For a reference copy of the document with all sections, see nature.com/documents/nr-reporting-summary-flat.pdf

Life sciences study design

All studies must disclose on these points even when the disclosure is negative.

Sample size	No sample size calculation was performed, and sample sizes were typically determined based on the availability of experimental data as it is a standard in the field.
Data exclusions	No sample was excluded, as reported in "Data acquisition and statistical analyses" section.
Replication	All the biological experiments were repeated no less than three times, unless otherwise stated. To assess reproducibility, statistical tests were performed and chosen based on the nature of the comparison being made and the standard tests used in the field. Sample independence, variance equality, and normality were assumed to be met although not explicitly examined. No experiment was excluded, and all experimental replicates are plotted in the graphs as individual data points. All experimental data is available in the "source data" supplementary file submitted with the manuscript. The number of experimental replicates and the statistical method used is reported in each figure legend and in "materials and methods" in "Data acquisition, data plotting and statistical analysis". Experiments such as the ones reported in Supplementary Figure 3 are standard in our lab, and were repeated and reproduced no less than five times, all attempts produced similar results, without excluding experiments. Experiment in Supplementary figure 9f was replicated two times, and the data for both replicates are in "source data" file. No experiment was excluded. Experiments such as the drug screen shown on Figure 3d and Supplementary Figure 6c were done once, but candidate hits were subsequently validated for no less than three times with similar results.
Randomization	For in vitro biological experiments, cell culture plates were randomly assigned to treatments in each biological experiment. Randomization described in "materials and methods", in " Data acquisition, data plotting and statistical analysis " section.
Blinding	Most biological imagine experiments were acquired and analysed automatically by a high content microscopy platform and software, and thus, data collection and analysis were blinded. For all the other experiments, data collection and analysis were blinded to the person collecting and analysing the data, and the samples were identified at the end of each experimental analysis.

Reporting for specific materials, systems and methods

We require information from authors about some types of materials, experimental systems and methods used in many studies. Here, indicate whether each material, system or method listed is relevant to your study. If you are not sure if a list item applies to your research, read the appropriate section before selecting a response.

Materials & experimental systems

n/a	Involved in the study
☐	☒ Antibodies
☐	☒ Eukaryotic cell lines
☒	☐ Palaeontology and archaeology
☒	☐ Animals and other organisms
☒	☐ Clinical data
☒	☐ Dual use research of concern
☒	☐ Plants

Methods

n/a	Involved in the study
☒	☐ ChIP-seq
☒	☐ Flow cytometry
☒	☐ MRI-based neuroimaging

Antibodies

Antibodies used

The following primary antibodies were used in this study: mouse monoclonal anti-p16-INK4a (JC8, Santa Cruz Animal Health) 1:1000 for Western Blot (WB); mouse monoclonal anti-p21 (CP74-P1484, Sigma) 1:1000 for WB; mouse monoclonal anti-IL1A (MAB200, R&D Systems) 1:100 for immunofluorescence (IF); mouse monoclonal anti-IL1B (MAB201, R&D Systems) 1:100 for IF and 1:1000 for WB; mouse monoclonal anti-IL8 (MAB208, R&D Systems) 1:100 for IF; mouse monoclonal anti-BrdU (555627, BD Biosciences,) 1:1000 for IF; monoclonal anti-b-Actin (A3854, Sigma) 1:40000 for WB.
The following secondary antibodies were used in this study: anti-mouse HRP (A2554, Sigma) 1:20000 for WB; goat anti-mouse Alexa Fluor 594 (A11032, ThermoFisher) 1:1000 for IF.

Validation

Antibody validation:
P16
https://www.scbt.com/es/p/p16-antibody-jc8
P21
https://www.sigmaaldrich.com/ES/es/product/sigma/p1484
IL1A
https://www.rndsystems.com/products/human-il-1alpha-il-1f1-antibody-4414_mab200
IL1B
https://www.rndsystems.com/products/human-il-1beta-il-1f2-antibody-8516_mab201
IL8
https://www.rndsystems.com/products/human-il-8-cxcl8-antibody-6217_mab208
b-actin HRP
https://www.sigmaaldrich.com/ES/es/product/sigma/a3854
BrdU
https://www.bdbiosciences.com/content/dam/bdb/products/global/reagents/flow-cytometry-reagents/research-reagents/single-color-antibodies-ruo/555627_base/pdf/555627.pdf
Alexa Fluor mouse
https://www.thermofisher.com/antibody/product/Goat-anti-Mouse-IgG-H-L-Highly-Cross-Adsorbed-Secondary-Antibody-Polyclonal/A-11032
mouse-HRP
https://www.sigmaaldrich.com/ES/es/product/sigma/a2554

Additionally, we validate the specificity of most antibodies using RNAi.

Eukaryotic cell lines

Policy information about cell lines and Sex and Gender in Research

Cell line source(s)

A549 NSCLC cell line and IMR90 female human fetal lung fibroblast cells were obtained from American Type Culture Collection (ATCC)

Authentication

None of the cell lines used were authenticated other than the ATCC authentication, but only low passage stocks from cells obtained from the ATCC were used in the experiments.

Mycoplasma contamination

All cells were routinely tested for mycoplasma with negative results. We have the policy of discarding mycoplasma positive cultures or cell line stocks. Mycoplasma contamination was tested using the Mycoalert Mycoplasma Detection Kit (Lonza).

Commonly misidentified lines
(See ICLAC register)

No commonly misidentified cell line was used.
